# Basic psychological need satisfaction and frustration in major depressive disorder

**DOI:** 10.3389/fpsyt.2022.962501

**Published:** 2022-09-20

**Authors:** Anou Pietrek, Maria Kangas, Reinhold Kliegl, Michael A. Rapp, Stephan Heinzel, Jolene van der Kaap-Deeder, Andreas Heissel

**Affiliations:** ^1^Social and Preventive Medicine, Department of Sports and Health Sciences, Faculty of Human Science, University of Potsdam, Potsdam, Germany; ^2^Centre for Emotional Health, School of Psychological Sciences, Macquarie University, Sydney, NSW, Australia; ^3^Division of Training and Movement Sciences, Research Focus Cognition Sciences, University of Potsdam, Potsdam, Germany; ^4^Social and Preventive Medicine, Department of Sports and Health Science, Intra-Faculty Unit “Cognitive Sciences”, Faculty of Human Science, and Faculty of Health Sciences Brandenburg, Research Area Services Research and e-Health, University of Potsdam, Potsdam, Germany; ^5^Department of Education and Psychology, Freie Universität Berlin, Berlin, Germany; ^6^Department of Psychology, Norwegian University of Science and Technology, Trondheim, Norway

**Keywords:** basic need satisfaction and frustration, depressive symptoms, clinical sample, need profiles, social environment

## Abstract

Basic psychological needs theory postulates that a social environment that satisfies individuals’ three basic psychological needs of autonomy, competence, and relatedness leads to optimal growth and well-being. On the other hand, the frustration of these needs is associated with ill-being and depressive symptoms foremost investigated in non-clinical samples; yet, there is a paucity of research on need frustration in clinical samples. Survey data were compared between adult individuals with major depressive disorder (MDD; *n* = 115; 48.69% female; 38.46 years, SD = 10.46) with those of a non-depressed comparison sample (*n* = 201; 53.23% female; 30.16 years, SD = 12.81). Need profiles were examined with a linear mixed model (LMM). Individuals with depression reported higher levels of frustration and lower levels of satisfaction in relation to the three basic psychological needs when compared to non-depressed adults. The difference between depressed and non-depressed groups was significantly larger for frustration than satisfaction regarding the needs for relatedness and competence. LMM correlation parameters confirmed the expected positive correlation between the three needs. This is the first study showing substantial differences in need-based experiences between depressed and non-depressed adults. The results confirm basic assumptions of the self-determination theory and have preliminary implications in tailoring therapy for depression.

## Introduction

Affective disorders are emotional disorders in which excessive sadness or severely elevated mood is exhibited over prolonged periods of time. The most common affective disorder is major depressive disorder (MDD). The World Health Organization names MDD as a leading cause of disability worldwide (1). Globally, over 250 million people (3.8% of the population) are currently affected by MDD, with 16% experiencing MDD at some point in their life (2). Phenomenologically, individuals with MDD show a persistent depressed mood, as well as a lack of drive and joy (3, 4). Given the relatively low level of remittance (5) and the high recurrence rate (6) of MDD, it is imperative to increase knowledge concerning the etiology and maintaining factors of MDD which can further improve treatment efforts.

The etiology of depression is multifactorial and involves the interaction of social, psychological, and biological factors. A growing volume of research has shown that MDD is associated with impairments in emotion regulation and cognitive control (7–11), which are associated with altered neural activation and connectivity patterns in fronto-parietal and fronto-limbic circuits (12–14). On a physiological level, recent literature examines the dynamics of the central and autonomic nervous systems in fear-related disorders and threat experience. The neurovisceral integration model of fear (NVI-f) (15) assumes a complex interplay between the central and peripheral nervous systems, which, in the case of a fear-inducing stimulus, passes through the prefrontal cortex, amygdala, and hippocampus to the heart and thus initiates fear reactions through sympathetic and parasympathetic projections (16). Here, the ventromedial prefrontal cortex (vmPFC) contributes to the learned distinction between threat and safety signals, in that the vmPFC supports positive affective processing of safety signals in conjunction with their implicit stress-relieving properties (17). As recent research shows, the ventromedial prefrontal cortex (vmPFC) plays a role in Pavlovian threat conditioning in humans not only in the extinction or reversal of previously acquired stimulus-outcome associations but also during threat acquisition (17–20). In patients with MDD, reduced volume and altered activity patterns were observed in the vmPFC (21–23). Importantly, recent work by Grahek et al. (24) highlights the need to investigate the motivational factors underlying depression. A leading theory concerning human motivation is the self-determination theory (SDT) (25), which details contexts and factors that contribute to both well-being and ill-being including psychopathology, such as MDD.

According to basic psychological needs theory (BPNT) (26), a subtheory within the broader SDT, there are three universally inherent basic psychological needs that are essential for individuals’ psychological growth, integrity, and well-being: autonomy, competence, and relatedness. The need for autonomy refers to the desire to feel volitional in the regulation of one’s behavior and experiencing one’s behavior as self-initiated; the need for competence refers to a perception of mastery through effectively interacting with the environment; and the need for relatedness refers to an experience of belonging and care with significant others (26). Well-integrated motivation, based on interest, joy or a sense of value, arises on the basis of fulfilled basic psychological needs (27). Some time ago, BPNT was extended to include the dimension of need frustration in addition to need satisfaction. Whereas, low psychological need satisfaction denotes the absence of need fulfillment, need frustration describes an experience of threat (28). Frustration of the need for autonomy involves the feeling of being pressured or even forced to think, feel, or act a certain way; frustration of the need for competence involves the experience of being defenseless and feeling like a failure; and frustration of the need for relatedness refers to an experience of being rejected or even ostracized (30). Need satisfaction and need frustration are asymmetrical related to each other, with high levels of need frustration always coinciding with low levels of need satisfaction, but low levels of need satisfaction not necessarily implicating a high level of need frustration. For example, although an individual might not feel connected to colleagues (i.e., low level of relatedness satisfaction), this person does not necessarily feel actively excluded by these colleagues (i.e., low level of relatedness frustration). While need satisfaction has been found to be especially conducive to adaptive outcomes (e.g., vitality), need frustration is implicated in ill-being and even psychopathology (28, 29). To illustrate, need frustration has been linked to stress (31), burn-out and eating symptomatology (32, 33), anxiety (34), and disengagement (35). Further, a recent study found need frustration being a partial mediator in the relation between emotion regulation and psychopathology (36).

Based on non-clinical samples, a large number of studies demonstrate a strong link between need frustration and depressive symptoms, showing the incremental (and sometimes sole) predictive value of need frustration over a lack of need satisfaction (30, 32, 37–39). A few studies have also examined the basic psychological needs in clinical samples of depressed individuals. A recent longitudinal study found that an increase in basic need satisfaction was associated with a decrease in depressive symptoms during the course of treatment (combined psychotherapy with medication) in a clinical sample with MDD (40). Similarly, Dwyer et al. (41) showed that a higher level of autonomy satisfaction during group therapy related to decreased depressive symptoms through a reduction in negative automatic thoughts in depressed adults.

In summary, the frustration of the basic psychological needs is proposed to be a general vulnerability factor for psychopathological symptom occurrence. However, disorder-specific research is needed to further investigate need-based functioning in the development, maintenance, and recovery of psychiatric disorders including MDD. However, to date, there is a notable paucity of research that has examined both need satisfaction and need frustration in a clinical sample with MDD. Accordingly, the aim of this study was to address this gap and to specifically examine whether individuals with depression show higher levels of need frustration alongside lower levels of need satisfaction when compared to individuals without depressive symptoms. On the basis of BPNT, we hypothesized that individuals without depressive symptoms would report being significantly more satisfied and less frustrated with regard to their needs than individuals with depressive symptoms on all three scales. We also expected that the difference between the two groups would be larger for frustration than satisfaction ratings for all three needs. Finally in an exploratory step, we added covariates of age, gender, and education to assess the generalizability of the main effects.

## Materials and methods

### Participants

This study was based on data of two adult samples from two recent German studies in order to comparatively evaluate the need domains (autonomy, competence, and relatedness) and dimensions (satisfaction and frustration) between clinically depressed and non-depressed adults.

The clinical sample with mild-to-moderate MDD was recruited from the SPeED study [Sport/Exercise Therapy and Psychotherapy—evaluating treatment Effects in Depressive patients; (42)]. The diagnosis of a mild or moderate depression episode in the SPeED study sample was confirmed using the Structured Clinical Interview, Axis I (SCID-I) (43, 44) according to the Diagnostic and Statistical Manual of Mental Disorders 4th Edition (DSM-IV) (3). Individuals with Becks Depression Inventory II (BDI-II) (45) scores < 14 [indicating no depressive symptoms; (46)] were excluded from the analyses (*n* = 4). The clinical sample at baseline/pre-treatment (T1) consisted of *n* = 115 (48.69% female) adults with a mean age of 38.46 years (*SD* = 10.46; range 21–65 years). The SPeED-Study protocol was approved by the local ethics committee of Charité Universitätsmedizin Berlin, Germany (No EA1/113/15). After detailed study description, written informed consent was obtained from all participants.

The non-clinical sample was recruited from the German Basic Psychological Need Satisfaction and Frustration Scale (BPNSFS) validation study (39), which included university students, working adults, and older adults. Data were collected in January and February of 2016. For the purposes of this study, participants were excluded if they reported depressive symptom scores greater than the cut-off value of 17 (*n* = 35) using the Center of Epidemiological Studies—Depression Scale (CES-D), and were older than 65 years (*n* = 103). The latter exclusion criterion was used to ensure that the samples between the two datasets were matched in age range, given the clinical sample was based on a sample younger than 65 years. The non-depressed sample consisted of *n* = 201 participants (53.23% female) with a mean age of 30.16 years (*SD* = 12.81; range 18–64). Participants provided written informed consent to take part in the study (39). The study protocol was approved by the Ethics Committee of the University of Potsdam (No. 41/2015).

Table 1 reports characteristics of the two samples. Significant statistical differences between the two samples were evident for depressive symptoms, age, and educational level.

**TABLE 1:** Participant characteristics by group.

	Non-depressed (*n* = 201)	Depressed (*n* = 115)	Group difference test
Gender (% female)	48.70	53.23	χ^2^_(1)_ = 0.44, *p* = 0.51
Age (mean, *SD*)	30.16, 12.81	38.46, 10.46	*t*_(277)_ = 6.24, ***p* < 0.001**
BDI (mean, *SD*)^a^	−/−	28.23, 7.32	*t*_(153.91)_ = 32.15, ***p* < 0.001**
CES-D (mean, *SD*)	7.51, 4.41	−/−	
Household income (mean, *SD*)	1430.41 (1403.75)	1678.07 (1226.44)	*t*_(241.73)_ = 1.59, *p* = 0.11
University entrance qualification (%)^b^	88.05	65.22	χ^2^_(1)_ = 13.97, ***p* < 0.001**
University grade (%)^b^	36.31	38.26	χ^2^_(1)_ = 0.11, *p* = 0.74

BDI, Becks depression inventory-II (45); CES-D, Center for Epidemiologic Studies Depression Scale (49).

^a^For group difference testing BDI and CES-D values were standardized on respective norm samples.

^b^University entrance qualification and University grade were considered as dichotomous variables. Bold values are significant p-values below 0.05.

### Main outcome measures

#### Depressive symptoms

In the two studies, depressive symptoms were assessed with different measurement instruments. In the clinical sample, the Becks Depression Inventory-II (BDI-II) (45) German version (47) was used. The BDI-II is a 21-item self-report questionnaire assessing depression symptom severity over a 2-week period, using a 4-point rating scale ranging from 0 (indicating absence of a symptom) to 3 (indicating a serve symptom occurrence), with elevated scores reflecting greater depressive symptom severity (total range of scores: 0–63). Sum scores 14–19 indicate mild depression, between 20 and 28, moderate depression, and between 29 and 63, severe depressive disorder (46). The German version of the BDI-II has demonstrated strong internal consistency (Cronbach’s alpha > or = 0.84) for clinical and non-clinical samples (48). Internal consistency in the present sample was also very good, α = 0.81.

In the non-clinical sample, depressive symptoms were measured using the Center for Epidemiologic Studies Depression Scale (CES-D) (49, 50). This scale assesses depressive symptoms in the general population by asking for the frequency of occurrence during the last 7 days (using a 4-point Likert scale with 0 = rarely or none of the time to 3 = most or all of the time). Scores range from 0 to 45, with scores above 17 indicating a depressive disorder. The German version has been found to have good internal consistency in the general population [Cronbach’s alpha = 0.90; (50)]. The reliability in the present sample was also adequate, α = 0.73. Table 2 reports relevant descriptive statistics and Cronbach’s α.

**TABLE 2:** Means (*M*), standard deviations (*SD*), and Cronbach’s alpha (α) by group for the six subscales of the Basic Psychological Need Satisfaction and Frustration Scale (BPNSFS).

Group	Scale	Dimension	*N*	*M*	*SD*	α
Group with major depressive disorder	Autonomy	Satisfaction	115	6.40	2.49	0.54
		Frustration		–9.65	3.26	0.78
	Competence	Satisfaction		7.57	3.37	0.83
		Frustration		–9.26	3.88	0.79
	Relatedness	Satisfaction		11.11	3.51	0.83
		Frustration		–5.52	3.16	0.64
Group without depressive symptoms	Autonomy	Satisfaction	201	11.26	2.43	0.68
		Frustration		–5.12	2.85	0.76
	Competence	Satisfaction		12.56	2.17	0.76
		Frustration		–2.83	2.43	0.65
	Relatedness	Satisfaction		13.97	1.85	0.61
		Frustration		–1.64	1.94	0.54

The score size ranges from –16 to +16 with a value of zero reflecting the perfect balance between satisfaction and frustration of a basic psychological need. Values in the positive spectrum show that positive satisfaction values exceed negative frustration values. Values in the negative spectrum show that negative frustration values exceed the level of need satisfaction.

#### Basic psychological need satisfaction and frustration scale

To assess basic psychological need satisfaction and frustration, the validated 24-item German version (39) of the BPNSFS (30) was used. Each of the three needs is operationalized with eight items, comprising two subscales, with items focusing on satisfaction or frustration of each need respectively (e.g., “I feel connected with people who care for me, and for whom I care.” —relatedness satisfaction and “I feel that people who are important to me are cold and distant toward me.” —relatedness frustration). Items are rated on a 5-point Likert scale, ranging from 1 (*completely disagree*) to 5 (*completely agree*). The internal consistency for each scale proved to be satisfactory, with Cronbach’s α ranging between 0.66 and 0.81 in the German sample (39) and between 0.64 and 0.89 in the original study by Chen et al. (30). The internal consistency in the present combined sample ranged between α = 0.74 for relatedness frustration and α = 0.89 for competence satisfaction. x

### Data analysis

Data analysis was conducted using International Business Machines Corporation (IBM) Statistical Package for the Social Sciences (SPSS) statistics software (Version 20) and the R system for statistical computing (R Version 4.1.3, R Studio 2022.02.1). We primarily used the following R packages: *dyplr* (51) to transform the data frame, *lme4* (52) for fitting the linear mixed effects models, *sjPlot* (53) for data visualization and graphics derived from the *ggplot* package (54).

The six subscale scores from the BPNSFS served as dependent measures in a Scale (3; autonomy vs. competence vs. relatedness) × Dimension (2; satisfaction vs. frustration) within-subject design. Group (2; non-depressed vs. depressed) was included as a between-subject factor. To maintain the same direction of effects for the six subscales (i.e., have them all correlate positively), the three frustration subscales were reversed (i.e., multiplied with –1). Adding four points to the scores of frustration subscales and subtracting four points from the scores of satisfaction subscales, scores ranged from 0 to –16 for the frustration subscales and 0 to +16 for the satisfaction subscales. Thus, higher scores indicate being better off in all six scales.

Inferential statistics were based on a linear mixed model (LMM) estimated with the *lme4* package in R (52). This approach makes it possible to estimate the main effects and interactions of group and dimension for each of the three need domains in a single analysis. In other words, we specified the group × dimension interaction as nested within the three levels of the factor scale, which is for autonomy, competence, and relatedness. All main effects and interactions of this Group (2) × Type of need (3) × Dimension (2) design were estimated as fixed effects. In a second step, this model was extended to check main effects of age, gender, and education and their moderation of group and dimension effects. In the random-effect structure, we estimated correlation parameters between the scores of the three scales and with the effect of dimension. This complex LMM was supported by the data. There was no need for parsimonious model selection (55). To test expected group difference in each of the six subscales, a *post hoc* LMM was performed and alpha error accumulation was taken into account with the Bonferroni method.

To assess the power of our analyses, we conducted sensitivity analyses using G*Power. Since we focus on group differences and directed hypotheses were formulated, the simulations are based on the *t*-test type with two independent means, one-tailed. Due to the required correction for multiple testing, the simulations also cover the range of smaller assumed alpha than α = 0.05. Sensitivity analysis revealed that even with a very low alpha of α = 0.001 and a desired power of (1–β) = 0.95, effects of moderate size (d = 0.55) are found with the available sample size.

## Results

Complete scores were available from 301 individuals (i.e., there were 1,806 observations). Table 3 lists fixed-effect estimates for main effects of group, dimension, age, gender, and a proxy for education (i.e., qualification for university entrance) as well as interactions of group with dimension and age. Table 3 also provides 95% credibility intervals for each effect, based on a likelihood profile and the appropriate cutoffs based on likelihood ratio tests, and p-values based on Wald statistics (i.e., estimate and standard error). Adding other interaction terms to the model did not significantly improve the goodness of fit.

**TABLE 3:** Fixed-effect estimates of three linear mixed models for each score of the basic psychological needs (Autonomy, Competence, Relatedness) including effects of the group (depressed vs. non-depressed), the dimension (satisfaction vs. frustration), as well as their interactions and covariate effects (age, gender, and university qualification).

Parameter	Estimate	CI	*P*-value
**Autonomy**			
Mean	0.40	[0.04, 0.79]	**0.034**
Group	2.32	[2.02, 2.77]	**<0.001**
Dimension	8.13	[7.98, 8.29]	**<0.001**
Age	0.01	[–0.01, 0.04]	0.268
Gender	0.28	[–0.56, –0.03]	**0.028**
University qualification (uniQ)	0.55	[0.19, 0.91]	**0.003**
Group × dimension	0.06	[–0.09, 0.21]	0.451
Group × age	0.01	[–0.02, 0.03]	0.574
**Competence**			
Mean	1.85	[1.45, 2.28]	**<0.001**
Group	3.08	[2.75, 3.57]	**<0.001**
Dimension	8.03	[7.87, 8.18]	**<0.001**
Age	0.05	[0.02, 0.08]	**<0.001**
Gender	0.49	[–0.76, –0.21]	**0.001**
University qualification (uniQ)	0.06	[–0.35, 0.46]	0.761
Group × dimension	–0.33	[–0.49, –0.18]	**<0.001**
Group × age	–0.03	[–0.06, –0.00]	**0.048**
**Relatedness**			
Mean	4.12	[3.77, 4.50]	**<0.001**
Group	1.78	[1.49, 2.21]	**<0.001**
Dimension	8.03	[7.87, 8.18]	**<0.001**
Age	0.02	[0.00, 0.05]	**0.041**
Gender	–0.32	[0.07, 0.56]	**0.010**
University qualification (uniQ)	0.34	[–0.02, 0.69]	0.056
Group × dimension	–0.21	[–0.37, –0.06]	0.007
Group × age	–0.02	[–0.05, 0.00]	0.096

Linear mixed model formula with effects nested in levels of Scale in *lme4* syntax in R: “Score ∼ 0 + Scale/(Group*(Dimension + age_c + UniQ)) + (0 + Scale + Dimension | Subj) Score ranges from –16 to +16. Mean estimates the mean of the respective Scale; Group estimates difference between non-depressed and depressed individuals; Dimension estimates difference between satisfaction and frustration; age_c is linear trend for age (centered); UniQ estimates the difference between individuals with and without university entrance qualification. Factor estimates are differences from mean of respective Scale. CI: 95% credibility intervals based on profiling of LMM estimates. *N* of observations = 1,806; *N* of individuals = 301. Bold values are significant p-values below 0.05.

**FIGURE 1:**
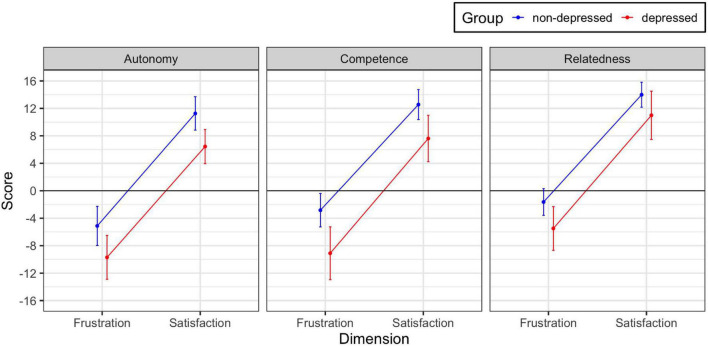
Observed group × dimension interactions for the three need domains. The interactions are significant for needs of competence and relatedness. Error bars are 95% confidence intervals.

Scores were higher for the satisfaction than the frustration dimension and for the non-depressed than the depressed group for each of the three need domains. Interactions between group and dimension were significant for competence and for relatedness. For both scales, the difference between the non-depressed and depressed groups was larger for the frustration than the satisfaction dimension (see Figure 1). A *post hoc* LMM showed that there were significant group differences for each of the six scales in the expected direction, even after Bonferroni Correction.

Further, the profile of means in Figure 1 exhibits large and significant group differences for the three scales; group differences between non-depressed and depressed were 4.7, 5.6, and 3.4 for autonomy, competence, and relatedness, respectively. In a subsequent exploratory *post hoc* analysis, the group difference on need of competence was significantly larger (*t* = 2.93) and the group difference for need of relatedness significantly smaller (*t* = –4.03) than the need for autonomy.

There were significant effects associated with covariates gender, education, and age. Scores were significantly larger for males than females for need of autonomy (1.7 vs. 1.3) and need of competence (3.4 vs. 2.6) and significantly larger for females than males for need of relatedness (5.4 vs. 4.7). There was also a significant difference between individuals with and without qualification for university entrance (1.8 vs. –0.2). None of the interactions of gender and education with group and dimension were significant. For age, needs for competence and relatedness increased significantly with age and, moreover, the age-related increase in competence was larger for depressed than non-depressed individuals (see Supplementary Figure 1). There were no significant interactions involving dimension.

The data supported the estimation of reliable variance components for the three scale scores and the dimension effect, as well as the associated correlation parameters (CPs) (i.e., individual differences; see Supplementary Table 1, left column and values below diagonal). The CPs among the scales were uniformly positive between 0.47 and 0.59, supporting an interpretation as a latent construct of basic psychological need. Note that the scale scores are averaged over satisfaction and frustration subscales. Two of the scales, autonomy and competence were significantly negatively correlated with the difference between satisfaction and frustration subscales. Thus, across this set of observations, it appears that the smaller the difference between the dimensions, the better off a person is. The corresponding zero-order correlations of scores are somewhat larger because they are not adjusted for differences due to group, sex, and age; the significant dimension-related CPs, however, are numerically slightly more negative than the corresponding zero-order correlations (–0.35 vs. –0.22; –0.25 vs. –0.22) because shrinkage correction increases the reliability of difference scores.

## Discussion

The present study investigated basic psychological need frustration and satisfaction in a sample of individuals with depression in comparison with a non-depressed control group for the three domains autonomy, competence, and relatedness. Individuals without depressive symptoms reported being significantly more satisfied and less frustrated than individuals with depressive symptoms with respect to all three needs. The results further showed that the group difference was significantly larger on the frustration than on the satisfaction dimension for competence and relatedness, illustrating the specific role of need frustration in the occurrence of psychopathological symptoms. Strengthening the benefit of implementing the frustration dimension in BPNT, this result aligns with previous research (28, 30, 39) and assumptions of BPNT. However, an interaction effect was not found for the need for autonomy in the present sample. Additionally, major moderation of the observed effects by age and educational status was not observed. The size of the group difference depends on both need domain and dimension. Although it might be assumed that scores are comparable across the three need scales, it is noteworthy that large differences for the size of group difference on the three scales emerged, with the largest group difference evident for competence experience and the smallest for relatedness experience.

The findings from this study make two novel, incremental advances in furthering our understanding of experienced basic need satisfaction and frustration in the context of MDD: First, competence frustration may play a critical role in at least maintaining symptoms in individuals with mild-to-moderate MDD. This concurs with psychopathology research findings and specifically aligns with depression-specific characteristics, such as a low self-efficacy-expectancy to the point of learned helplessness (56–60). Second, unlike the other two need domains for individuals with elevated depressive symptoms, the experience of relatedness also shows less satisfaction and more frustration in comparison to non-depressed adults, although it remains relatively stable in a mild to moderate Major Depressive Episode. Accordingly, not all need domains are affected equally. A related assumption that has received less attention to date is that not only a high but also a balanced satisfaction of needs is presumed to be conducive to well-being and growth-processes, with balanced need satisfaction referring to similar scores on need satisfaction across the three needs (61). Although satisfaction of one need typically parallels the satisfaction of the other needs, this is not always the case. Ryan and Deci (62) state that basic psychological needs can be experienced in conflict, even if not being inherently contradictory. Unbalanced need satisfaction indicates a conflictual experience in that the satisfaction of one need may be foregone in favor of another (e.g., excessive focus on externally imposed performance). Initial research results support this proposition and studies have shown that both need satisfaction and balanced need satisfaction were predictive of well-being (63–65). Thus, in addition to the score of the individual scales, the between-need-balance merits further investigation. One possible explanation for the present findings would be a tendency to accept a loss in the needs for autonomy and competence while a certain experience of relatedness is maintained. Autonomous behavior is sometimes accompanied by a detachment from a particular mode of relationship (e.g., interpersonal dependence). Interpersonal dependency on the other hand has been cited as an individual vulnerability factor for depression (66–68). Accordingly, it is conceivable that especially the conflictual experience of basic psychological needs (e.g., when an individual can only fulfill his/her need for relatedness by sacrificing his/her autonomy) leads to the inhibition of growth processes, maladaption and potentially ill-being. However, further longitudinal research is needed to determine whether this is a depression- and/or course-specific pattern.

### Limitations and direction for future research

The findings from this study need to be interpreted in relation to some limitations. Given the cross-sectional study design, the findings are limited to group-comparison at a specific timepoint. Changes in the need profiles during the course of disease or treatment were not investigated. This study also focused on a clinical sample with mild-to-moderate MDD compared to a non-depressed sample, while not representing the broader mental health status of the control group nor including participants with severe major depressive episodes. Further, individuals in the non-depressed sample overall had a higher level of education and were younger than the clinical sample. Due to the large age range of the present sample and the observation that age itself has an influence on the experience of basic psychological needs future research could, therefore, incorporate a longitudinal design thereby employing a matched control group in addition to a clinical sample displaying mild-to-severe MDD.

Further, with the nested LMM, we do not provide direct tests between the different needs. The decision for the nested LMM was motivated by the assumption that, for example, the degree of autonomy satisfaction is not comparable with the degree of competence satisfaction. A meaningful avenue for future research is to consider how such comparisons might be achieved and broaden the theoretical scope of BPNT. Assuming that the scales can be compared with each other in terms of their magnitude, future research with the BPNSFS could contain nested specification of group × dimension with tests of interactions involving each scale. Group-specific need profiles have great potential to further elucidate psychological disorder symptom severity, progression, and processes and to enable targeted, profile-based interventions. Thereby, need profiles should be investigated in diverse samples on the continuum of well-being to ill-being and across various psychological disorders. Finally, future research is warranted to examine the specific role of the needs in relation to other factors found to be important in depression, such as emotion regulation (8, 36), cognitive control (7, 10), and neural deficits (13, 14, 69).

## Conclusion and practical implications

The study of need frustration as a separate dimension from need satisfaction has great potential to investigate threat experience and its motivational and behavioral consequences underlying psychopathology (i.e., MDD). This is the first study to demonstrate that there is a substantial difference between non-depressed adults and adults with mild-to-moderate depression in terms of their need satisfaction and need frustration. As hypothesized, people with MDD show lower satisfaction and higher frustration of the basic psychological needs for autonomy, competence and relatedness. Assuming that need frustration plays a transdiagnostic role accounting for a diversity of pathological symptoms, the question remains open whether disorder-specific need-based dynamics can be found that allow a deeper understanding and more targeted interventions. The present findings highlight the potential benefits of examining the amount as well as the balance of basic psychological need satisfaction and frustration in disorder-specific samples to further investigate compensatory, substitute, and reactive processes associated with need frustration as well as experienced conflict between the needs in detail.

Specifically, our findings suggest that people experiencing MDD would profit from social environments supporting all basic psychological needs with a particular focus on competence experience. For example, this could take the form of psychotherapy (5, 70), in which the meaning of failures could be discussed or exercise therapy (71–73), in which successes can be celebrated together. An assumption that would be consistent with the present findings is that individuals with MDD experience their psychological needs as being in conflict with each other. Then, it would also be conceivable to work with patients on a perceived conflict between basic psychological needs so that a balanced satisfaction of needs can be achieved.

## Data availability statement

The raw data supporting the conclusions of this article will be made available by the authors, upon reasonable request.

## Ethics statement

The studies involving human participants were reviewed and approved by Ethics Committee of Charité Universitätsmedizin Berlin, Germany, and Ethics Committee of the University of Potsdam. The patients/participants provided their written informed consent to participate in this study.

## Author contributions

AH, AP, and RK made substantial contributions to conception and design and wrote the first draft of the manuscript. AP and RK carried out data analysis. AH, AP, MK, JK-D, and RK interpreted the data. All authors were involved in revising the manuscript critically for important intellectual content and provided final approval of the version to be published.
