# Supplementary material for: Basic psychological need satisfaction and frustration in major depressive disorder
Source: Front Psychiatry. 2022 Sep 20;13:962501. doi: 10.3389/fpsyt.2022.962501 (PMC9530199; doi:10.3389/fpsyt.2022.962501)
Supplement: Supplementary file 1 [file Table_1.DOCX]

Supplementary Table

Supplementary Table 1. LMM variance components (*VC*) and correlation parameters (*CP;* below diagonal) and zero-order correlations (*r;* above diagonal) for scale scores

|  | **VC** | **CP \ r** | | | | |
| --- | --- | --- | --- | --- | --- | --- |
| **Component** |  | **Autonomy** | **Competence** | **Relatedness** | **Dimension** |  |
| Autonomy | 1.91 | 1.00 | **0.73** | **0.60** | **-0.25** |  |
| Competence | 2.16 | 0.59 | 1.00 | **0.65** | **-0.22** |  |
| Relatedness | 1.79 | 0.47 | 0.58 | 1.00 | **-0.15** |  |
| Dimension | 0.58 | -0.36 | -0.25 | -0.07 | 1.00 |  |

*Note*. Autonomy, Competence and Relatedness are scale scores;
Dimension is effect score (i.e., satisfaction – frustration); residual variance is 1.63. CP \ r for Dimension and Relatedness are not significantly different from zero.
