# Supplementary figures and images for: Basic psychological need satisfaction and frustration in major depressive disorder
Source: Front Psychiatry. 2022 Sep 20;13:962501. doi: 10.3389/fpsyt.2022.962501 (PMC9530199; doi:10.3389/fpsyt.2022.962501)

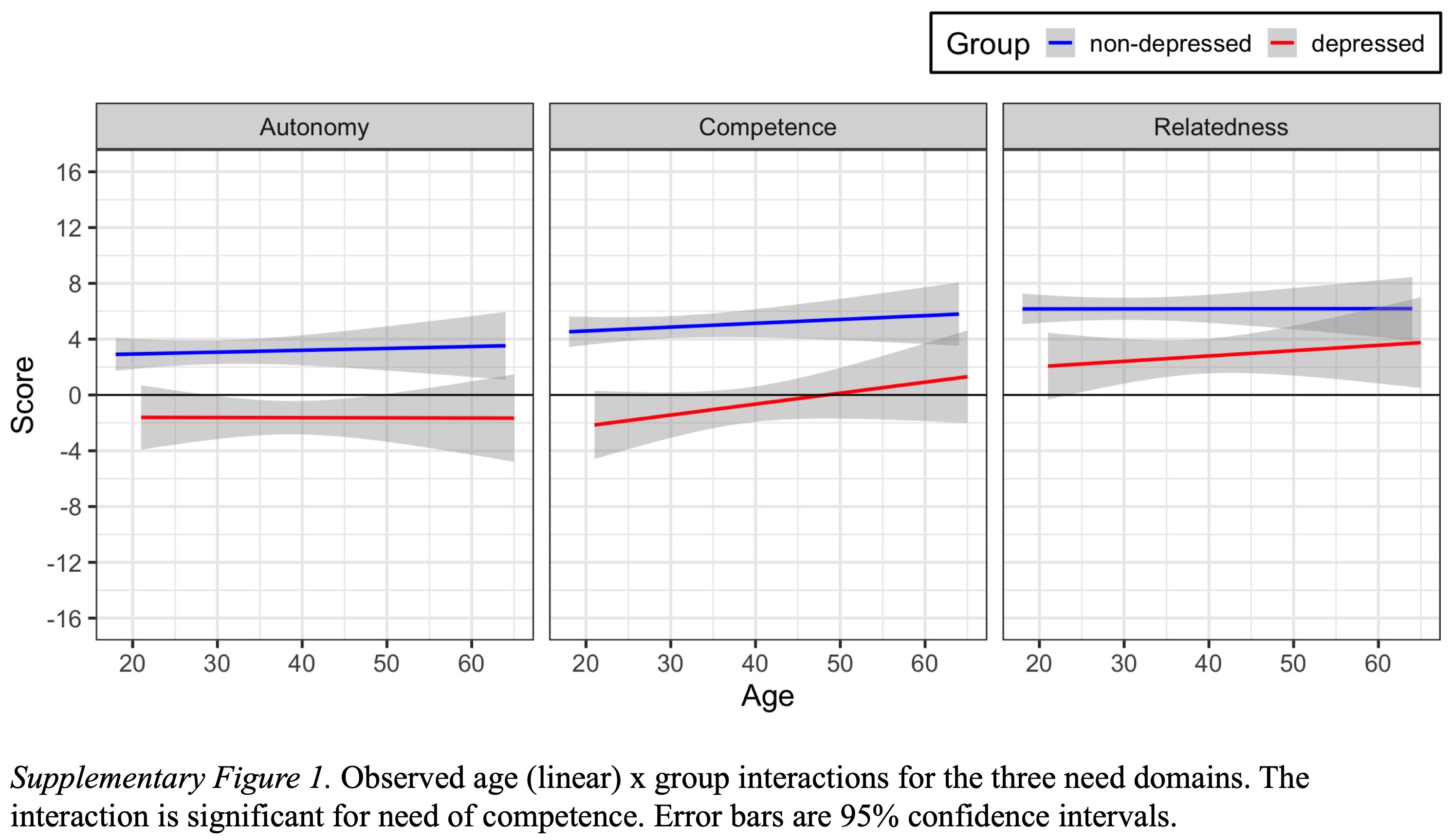

Supplement: Supplementary file 2 [file Image_1.JPEG]
